# The mother–daughter Shared Agency in Weight Management Scale (SAWMS): development, validation, and implications for body dissatisfaction

**DOI:** 10.1186/s40337-023-00747-6

**Published:** 2023-02-20

**Authors:** Jianmin Shao, Esther S. Chang, Yuying Tsong, Chuansheng Chen, Jessica L. Borelli

**Affiliations:** 1 Department of Psychological Science, 4201 Social and Behavioral Sciences Gateway, University of California, Irvine, Irvine, CA 92697-7085 USA; 2 Social and Behavioral Sciences Division, Soka University of America, Aliso Viejo, CA USA; 3 Department of Human Services, California State University, Fullerton, Fullerton, CA USA

**Keywords:** Maternal control, Maternal autonomy support, Mother–daughter relationship, Shared agency, Body dissatisfaction, Weight management

## Abstract

**Background:**

Much research suggests that mothers play an important role in shaping daughters’ body image, yet less is known about how mother–daughter relationship dynamics in weight management affect daughters’ body dissatisfaction. The current paper described the development and validation of the mother–daughter Shared Agency in Weight Management Scale (SAWMS) and examined its associations with daughter’s body dissatisfaction.

**Methods:**

In Study 1 (*N* = 676 college students), we explored the factor structure of the mother–daughter SAWMS and identified three processes (*control, autonomy support,* and *collaboration*) whereby mothers work with daughters in weight management. In Study 2 (*N* = 439 college students), we finalized the factor structure of the scale by conducting two CFAs and assessing the test–retest reliability of each subscale. In Study 3 (same sample as Study 2), we examined the psychometric properties of the subscales and their associations with daughters’ body dissatisfaction.

**Results:**

Combining results from EFA and IRT, we identified three mother–daughter dynamics in weight management—maternal *control*, maternal *autonomy support*, and maternal *collaboration*. However, based on various empirical results indicating poor psychometric properties of the maternal collaboration subscale, we removed it from the mother–daughter SAWMS and only evaluated the psychometric properties of the remaining two subscales (i.e., control and autonomy support). They explained a significant amount of variance in daughters’ body dissatisfaction over and above the effect of maternal pressure to be thin. Maternal *control* was a significant and positive predictor of daughters’ body dissatisfaction; maternal *autonomy support* was a significant and negative predictor.

**Conclusions:**

Results suggested that maternal control in weight management was associated with daughters’ increased body dissatisfaction, whereas maternal autonomy support in weight management was associated with daughters’ lower body dissatisfaction. These specific ways in which mother work with daughters in weight management provide nuances in understanding young women’s body dissatisfaction. Our SAWMS offers new ways to examine body image among young women through the mother–daughter relationship dynamics in weight management.

## Plain English summary

The current study described the procedure of developing a new measurement—the mother–daughter Shared Agency in Weight Management Scale (SAWMS). This scale aims to measure the different ways in which mothers work with their cisgender daughters in weight management. Based on self-reported survey data from cisgender female college students, we identified two ways whereby mothers work with their daughters in weight management—maternal control and maternal autonomy support. To better understand these mother–daughter dynamics, we also examined their relations with daughter’s body dissatisfaction. We found that daughters whose mothers were more controlling when it comes to weight management reported higher levels of body dissatisfaction. On the other hand, daughters whose mothers were more autonomy-supportive in weight management reported lower levels of body dissatisfaction. Our results have important implications for understanding how mother–daughter relationship dynamics in weight management may contribute to the development of body image and perceptions among young women.

## Background

Researchers and health professionals have long been interested in understanding the development and prevention of eating disorders. In this vein, much attention has been paid to the internalization of body weight ideals, body dissatisfaction, and desire for thinness among young women (e.g., [46]). As is intuitively obvious, social pressures to be slim can affect youth perceptions and internalization of ideal body image [8] (Levine et al. 1994). Parents, especially mothers, may also play significant roles in daughters’ eating and body weight management, for better and for worse [23, 36]. Indeed, for young women in particular, mothers’ support and understanding in weight management might be protective against the judgments and pressure from external social environments [42]. On the other hand, mothers’ control and policing on body image might exacerbate young women’s body dissatisfaction, which, in turn, may have consequences for disordered eating [14].

Despite the significance of maternal influence on daughters’ body image, research has not yet identified the processes through which mothers interact with daughters in weight management issues. Aligning with the recent call from scholars of eating disorders to center mother–daughter dynamics in understanding young women’s body image and disordered eating [7], we developed and validated a novel measure examining how mothers work with their daughters in weight management—the mother–daughter Shared Agency in Weight Management Scale (SAWMS). Guided by the framework of shared and non-shared agency, which sheds light on how parents and youth interact with each other in pursuing life directions and negotiations [11], we identified two ways in which mothers work with their daughters in weight management: maternal control and maternal autonomy support. We also examined the implications of these mother–daughter dynamics in weight management for daughters’ body dissatisfaction.

## Maternal socialization of body image and the mother–daughter relationship dynamics

Given the higher risk of eating disorders among cisgender women than cisgender men [12] and the important health implications of maternal influence [35], much research has focused on how mothers affect body image and weight concerns among young cisgender women. After all, women from different generations are, in one way or another, socialized in phallocentric societies where patriarchal values are transmitted, sustained, and learned through interactions in everyday life [1]. As such, the relational dynamics between mothers and daughters may be a potential site for both risks (e.g., maternal judgment) and opportunities (e.g., collectively contestation of patriarchal ideals) for young women’s body images and weight management. For example, in samples of girls and young women with diverse ethnic backgrounds, maternal comments and criticisms regarding weight are significant predictors of daughters’ eating pathology [36, 49]. Similarly, a qualitative study with Israeli women reported that many participants first received negative comments on their body shape and weight from their mothers [33]. These judgmental messages from mothers coupled with the readymade sociocultural system rife with patriarchal ideals about women’s bodies and the so-called fat talk can be devastating to many young women, negatively affecting their body perceptions and eating behaviors [16]. Even though these maternal messages can at times be encouraging and without malicious intentions, previous studies show that they are significantly associated with daughters’ increased body dissatisfaction even after controlling for BMI [4, 25], for these messages might give daughters the impression that their weight is unacceptable [27].

Direct maternal messages, however, do not always need to be maladaptive. Much existing literature examining maternal influence on daughters’ body image tends to focus more on risk factors (e.g., controlling parenting behaviors) than on the positive mother–daughter relationship dynamics [7]. Some studies with diverse samples from the United States have shown that higher levels of mother–daughter relationship quality were associated with better body image among both adolescent girls and college-aged women [24, 42],other studies with a majority of White participants have indicated that parental-autonomy support were protective against youth’s body dissatisfaction [22, 37]. A few studies with mostly White adolescent girls and college-aged women, however, did not find maternal support and mother–daughter closeness to be protective against daughters’ body image concerns [14, 34], yet, these studies often measured general support or relationship qualities rather than specific mother–daughter dynamics with respect to in weight management.

Importantly, what seems to be missing in these previous investigations is how mothers might work with their daughters on weight management and its consequences. The identification of these social processes is crucial because it can lead to more detailed answers for how mother–daughter dynamics in weight management may be harmful and/or healthy for the development of daughters’ body image, potentially informing preventive efforts related to disordered eating. Given that both parents and youths are capable of influencing each other [11], the shared agency between mothers and daughters in weight management and its implications for daughters’ body dissatisfaction are worth investigating.

## Shared agency between youth and parents

The framework of shared and non-shared agency [11], which captures various ways of parent-youth interaction in life pursuit, originated from literatures on parenting and educational goal attainment among emerging adults. Previous studies have indicated that shared agency between parents and college-aged youth can be promoted in three ways [10, 11]. Parents can *support* youth’s efforts in life pursuit by granting greater autonomy as youth become mature enough to insert more agency in decision-making. Parents and youth might *collaborate* with each other so that they can make joint decisions, to the extent that youth’s autonomy is not threatened and their competence is not questioned. Parents can also *accommodate* youth and yield to the way youth handle goals. Youth will perceive it as shared agency when they feel that their parents are invested and interested in their success. Non-shared agency between parents and youth refers to parent–child dynamics wherein one party is disengaged from goal pursuit [11]. Parents might be *uninvolved* when youth are motivated in their own pursuit without parents being a secure base of support. Parents might also be *directing* or *overinvolved* when they try to exert control over youth’s behaviors and efforts. Importantly, these distinct parent-youth dynamics in which both parties show differential levels of agency have implications for mental health and educational adjustment among youth and emerging adults. Much parenting research suggests that parental autonomy support and shared agency with youth seem to be beneficial for psychosocial development, whereas parental non-shared agency with youth (e.g., controlling) might constrain positive psychological and educational adjustment [11, 30, 38].

When it comes to daughters’ body image and weight, which are of particular concern for women themselves due to experiences in patriarchal societies, mothers might react in various ways given their own positionalities as women and caregivers. For example, mothers could be perceived by daughters as *supportive* when they offer advice while providing daughters with freedom to explore body shape and weight without any judgement, or as *accommodating* when they take a step back to let daughters follow their own heart. Mothers could also be perceived as *collaborative* when they help daughters achieve goals while respecting daughters’ own decisions without too much control. Finally, mothers could be *controlling* when they become overinvolved to the extent that daughters lack agency in making decisions about their own bodies.

Considering that the effects of parent–child relationship dynamics are well-documented in domains such as developmental psychopathology [5] and educational adjustment [11], they could also have profound implications for body image. Yet, very little research has shed light on how shared and non-shared agency between college-aged youth and parents might affect youth’s body image. This paucity of research might be due in large part to the unavailability of validated measures. Thus, we aim to develop and validate a scale measuring mother–daughter relationship dynamics in weight management. It could be that daughters need mothers’ experiences and advice so that collaboration helps navigate difficulties in eating and external pressure for perfect body image. On the other hand, it could be that body image is a personal matter so that autonomy support is desired, whereas any kind of maternal control and directing are detrimental. As such, we also examine the associations between mother–daughter dynamics in weight management and daughters’ body dissatisfaction.

## Present studies

In three studies, we aimed to delineate the development, validation, and initial assessment of psychometric properties of the mother–daughter Shared Agency in Weight Management Scale (SAWMS). We rely on young women’s self-reports of experiences with their mothers and their current body image and dissatisfaction. In Study 1, based on survey responses of a group of young college women, we describe the development of the scale as well as the processes of item selection and reduction, using exploratory factor analysis (EFA) and item response theory (IRT). In Study 2, using a separate sample of young college women, we finalized the factor structure of the scale. Specifically, we conducted confirmatory factor analyses (CFA; a two-factor model and a three-factor model) and assessed the test–retest reliability of each subscale using six-month follow-up data from a subsample. In Study 3, using the same baseline sample as Study 2, we examined psychometric properties of the subscales—convergent, divergent, discriminant, and incremental validities (e.g., associations between each subscale and other relevant measures; using the subscales predicting body dissatisfaction over and above an existing measure).

## Study 1: Development and item selection

The concept of shared and non-shared agency with parents on which the SAWMS is based was initially developed in the domain of educational goal attainment [11]. The original items measuring parent–child dynamics in educational attainment were based upon existing measures of parental autonomy support, parenting styles, and parental psychological and behavioral control (e.g., [13, 44]). In-depth focus group interviews with immigrant mothers of college students were then conducted to refine the items for relevance to college students from interdependent family backgrounds [9]. To adapt these items for young women’s weight management, the second and third authors—one a developmental psychologist specializing in adolescent development (who also developed the scale of shared and non-shared agency with parents in educational attainment [11]; and the other a counseling psychologist specializing in eating disorders—revised them for relevance to weight management issues and assessed their face validity. Due to the various degrees of parental involvement in youth’s development (e.g., from overcontrol to complete uninvolvement), the initial items included four ways in which daughters may perceive how their mothers work or not work with them in weight management: maternal *directing*, maternal *collaboration*, maternal *support*, and maternal *accommodation*. Given that the shared agency framework has not been previously applied to the domain of weight management and body image, the current study aimed to explore the factor structures of the scale using EFA and to refine the selection of final items using IRT.

## Method

### Participants

The current sample consists of 676 US born and raised cisgender college-enrolled women who were at the age between 18 to 24 years old (*M* = 20.31, *SD* = 1.40) from a public research university in Southern California. The original dataset included cisgender men as well as cisgender women who did not have a mother figure in their lives; we excluded them in the current study due to our focus on the mother–daughter relationship dynamics. We also deleted 10 cases that had no data on the SAWMS. The current sample is ethnically diverse: 253 (37.4%) Asian and Pacific Islander Americans, 243 (35.9%) Latinx Americans, 90 (13.3%) European Americans, and 90 (13.3%) who identified as biracial or other minority ethnicities (i.e., African American, Native American, Middle Eastern American).

### Procedure

After obtaining approval from the Institutional Review Board at the principal investigators’ institutions, an online survey was distributed through the Human Subject Pool at a public research university in Southern California during Spring 2019. Students who were over 18 and enrolled in the subject pool were eligible for participation in exchange for half an extra class credit. They completed the 15-item mother–daughter SAWMS in a survey asking about their eating habits and behaviors as well as their mothers’ behaviors and attitudes toward their weight management. Specifically, participants were asked to indicate the extent to which they agree or disagree (1 = *Strongly disagree*, 6 = *Strongly agree*) with the statements on how their mothers work with them in weight management.

### Data analysis

We first conducted an EFA using maximum likelihood estimation with promax rotation to explore the underlying factor structure of mother–daughter SAWMS. Given that all items were normally distributed (skewness ranged from − 0.88 to 0.88; kurtosis ranged from 1.72 to 3.00), we chose maximum likelihood estimation to align with best practice [18]. We decided to use promax rotation for its conceptual parsimony and oblique characteristic, which allows factors to be correlated [15]. Among the 15 items, six had no missing data, and nine had only one or two cases missing. Due to very few missing data in the sample (i.e., less than 3%), we ran the EFA with mean replacement [47]. Given that a few items did not load on our theorized factor and thus resulting in unbalanced number of items in each subscale (see details in Results and Table 1), we used IRT to refine results based on EFA. Considering the polytomous and ordered characteristics of the items, we used Graded Response Model (GRM) for item calibration [17]. To assess the relative IRT model-data fit for each subscale, we first used generalized structural equation modeling in Stata 15 to estimate (1) a parsimonious GRM that constrained the slope to be the same for all items and (2) a full GRM that specified unique slopes for each item. We then compared the fit of these two nested models using a likelihood ratio test [17]. Finally, we relied on both empirical evidence (i.e., EFA and IRT results) and theoretical considerations to select items that had maximum precision while maintaining adequate content coverage for each subscale [28, 32]. Specifically, we considered the following criteria: (1) high item discrimination, (2) high item information, (3) reasonable spread of difficulties, (4) high item-subscale correlation, and (5) adequate content coverage for each subscale.

**Table 1:** EFA Factor Loadings for Perceived Mother–Daughter Shared Agency in Weight Management

SAWM items	Factor 1	Factor 2	Factor 3
	Directing	Support/Accommodation	Collaboration
1. My mom wants me to be a certain body weight	**0.69**	− 0.12	− 0.06
2. My mom tries to make me eat more or less because of my body weight	**0.77**	0.12	− 0.07
3. My mom attempts to control my eating portions because of my weight	**0.71**	− 0.01	0.10
4. My mom nags at me about how I am managing my weight	**0.86**	0.003	− 0.15
5. My mom talks with me about my weight*	**0.62**	− 0.05	0.22
6. My mom works with me on my body weight issues	0.07	0.01	**0.78**
7. My mom negotiates with me on how I manage my body weight*	**0.61**	0.12	0.23
8. My mom works together with me in managing my body weight	0.02	− 0.19	**0.93**
9. My mom is very supportive of how I manage my weight	− 0.19	**0.58**	0.24
10. My mom encourages me to be the weight I want to be	0.09	**0.73**	0.15
11. My mom helps me to be the weight I want to be*	0.02	0.20	**0.66**
12. My mom just wants me to be happy about my body weight	− 0.09	**0.67**	0.10
13. My mom can let go of her own issues with my body weight	0.14	**0.45**	0.03
14. My mom does not feel responsible for managing my weight	0.10	**0.65**	− 0.32
15. My mom feels like my body weight is my own business	− 0.21	**0.60**	− 0.06
Cronbach's alpha	0.86	0.80	0.83

Factor loadings in bold indicate that items loaded on the corresponding subscale

*N* = 676. Items 1–4 were theorized to indicate maternal directing; 5–8 maternal collaboration; 9–11 maternal support; 12–15 maternal accommodation. *Items that loaded on the factor other than the theorized one. The order of the items in the executed survey was randomized for participants

## Results

### EFA results

Preliminary tests indicated that the data were suitable for factor analysis, *KMO* = 0.904, Bartlett’s test of sphericity: χ^2^ (105) = 4629.64, *p* < 0.001. Examination of the scree plot combined with the rule of eigenvalues greater than 1 indicated that a three-factor solution best fit the data, and the EFA using maximum likelihood estimation with promax rotation extracted three factors onto which all 15 items loaded. Rotated factor loadings ranged from 0.606 to 0.864 for maternal *directing*, 0.450 to 0.728 for maternal *support/accommodation*, and 0.655 to 0.972 for maternal *collaboration*. The three factors accounted for 63.03% of the common variance. Correlations among the factors ranged from − 0.59 (directing with support/accommodation) to 0.15 (directing with collaboration). The first factor (directing) accounted for 33.61% of the common variance. The second factor (support/accommodation) accounted for 22.56% of the common variance. The third factor (collaboration) accounted for 6.85% of the common variance. Factor loadings and Cronbach's alphas are presented in Table 1. As can be seen, alphas of all three subscales for the current sample were high (i.e., alphas ≥ 0.80).

### IRT calibration

For the maternal directing subscale, the likelihood ratio test indicated that a fully specified GRM with each item having a unique slope yielded better model fit than the reduced GRM with a single slope across all items, *X*^*2*^ (*df* = 5) = 83.3, *p* < 0.001. For the maternal support/accommodation subscale, the likelihood ratio test also showed that the fully specified GRM had better fit than the constrained GRM, *X*^*2*^ (*df* = 5) = 100.47, *p* < 0.001. For the maternal collaboration subscale, however, the fully specified GRM was not a better fit to the data than the constrained GRM for, *X*^*2*^ (*df* = 2) = 5.29, *p* = 0.07, which indicates that the three items indicating maternal collaboration were not significantly different in their ability to discriminate among respondents. Considering that the three items indicating maternal collaboration have strong face validity and high factor loadings (all > 0.65, see Table 1), we decided to examine their item properties.

### Item properties and final selection

**Fig. 1:**
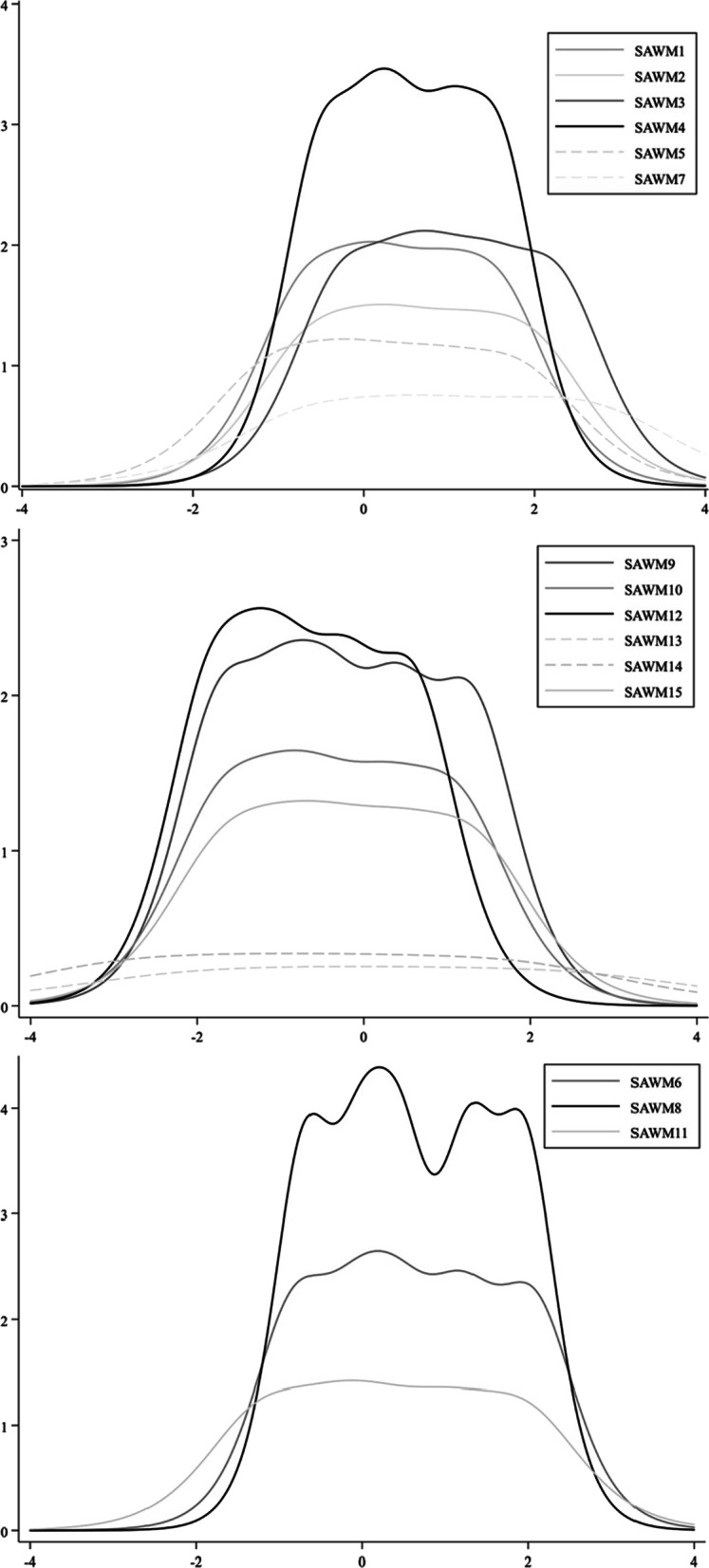
Item Information Curves for SAWMS Subscale Items.* Note* Panels from the upper to the lower represent subscales of control, autonomy support, and collaboration in weight management, respectively. X-axis reflects item difficulty; y-axis reflects item information. Items included in the final subscale are in solid lines. Items excluded due to low discrimination are in dash lines

IRT parameter estimates and item-subscale correlations are presented in Table 2. For maternal directing subscale, the discrimination parameter estimates ranged from 1.55 to 3.34, indicating high (1.35–1.69) to very high discrimination (> 1.7; [2]). The difficulty parameters had appropriate spread (− 0.70 to 2.75); the majority of them were above 0, suggesting that the item set as whole was most efficient in discriminating among individuals who experienced higher than average levels of maternal control. Considering that items 5 and 7 have relatively lower discriminations, item information, and item-subscale correlations than the rest of the items and that they were not originally theorized as maternal directing, we decided to exclude them and rename this subscale as *maternal control* to reflect mothers’ controlling behaviors in daughters’ weight management. Item information curves are shown in Fig. 1.

**Table 2:** Item Response theory parameter estimates and item-subscale correlation for the SAWM Scale

Subscale	Item	Item parameter estimates						Item-subscale correlation
		*a*	*b*_*1*_	*b*_*2*_	*b*_*3*_	*b*_*4*_	*b*_*5*_	
Maternal control	SAWM1*	**2.54**	**− 0.70**	**− 0.08**	**0.28**	**0.95**	**1.58**	**0.79**
	SAWM2*	**2.18**	**− 0.62**	**0.03**	**0.45**	**1.19**	**1.94**	**0.76**
	SAWM3*	**2.60**	**− 0.27**	**0.45**	**0.86**	**1.49**	**2.26**	**0.78**
	SAWM4*	**3.34**	**− 0.51**	**0.08**	**0.40**	**1.02**	**1.59**	**0.84**
	SAWM5	1.96	− 1.10	− 0.40	0.04	0.85	1.80	0.75
	SAWM7	1.55	− 0.63	0.35	0.96	2.02	2.75	0.66
Maternal autonomy support	SAWM9*	**2.79**	**− 1.73**	**− 0.98**	**− 0.42**	**0.41**	**1.31**	**0.78**
	SAWM10*	**2.29**	**− 1.71**	**− 1.01**	**− 0.53**	**0.28**	**1.11**	**0.76**
	SAWM12*	**2.86**	**− 1.86**	**− 1.34**	**− 0.92**	**− 0.21**	**0.61**	**0.78**
	SAWM13	0.89	− 2.02	− 0.83	− 0.03	0.90	2.45	0.57
	SAWM14	1.03	− 2.85	− 1.61	− 0.72	0.30	1.77	0.60
	SAWM15*	**2.05**	**− 1.64**	**− 0.93**	**− 0.37**	**0.43**	**1.31**	**0.76**
Maternal collaboration	SAWM6*	**2.94**	**− 0.80**	**− 0.03**	**0.42**	**1.20**	**2.06**	**0.87**
	SAWM8*	**3.83**	**− 0.68**	**0.02**	**0.45**	**1.30**	**1.96**	**0.88**
	SAWM11*	**2.13**	**− 1.19**	**− 0.36**	**0.15**	**1.03**	**1.95**	**0.85**

Coefficients in bold indicate that items were retained in the version of the mother-daughter SAWMS after IRT analysis

a = discrimination parameter; b = estimated item difficulties corresponding to a 50% chance of choosing the response that is ≥ the subscript + 1; *selected item for inclusion in final subscales

For maternal support/accommodation subscale, the discrimination parameter estimates ranged from 0.89 to 2.86, suggesting moderate (0.65–1.34) to very high discrimination (> 1.7; [2]). The difficulty parameters had appropriate spread (− 2.85 to 2.45), more than half of them were below 0, suggesting that the item set as whole was most efficient in discriminating among individuals who experienced lower than average levels of maternal support/accommodation. Considering that items 13 and 14 had lower discriminations and item-subscale correlations and much lower information than the rest of the items, we decided to exclude them. Given that the final four items consisted of items previously theorized as maternal support and maternal accommodation, we renamed this subscale as *maternal autonomy support* to better reflect its construct. Item information curves are shown in Fig. 1.

For maternal collaboration subscale, all items had very high discrimination (2.13 to 3.83; [2]). The difficulty parameters had appropriate spread (− 1.19 to 2.06), more than half of them were above 0, suggesting that the item set as whole was most efficient in discriminating among individuals who experienced higher than average levels of maternal collaboration. Given that all three items showed high discriminations, item information, item-subscale correlations, and appropriate spread of difficulty, we decided to keep all of them. Item information curves are shown in Fig. 1.

## Study 2: Confirmatory factor analyses and test–retest reliability

Guided by the theoretical framework of shared agency between parents and youth [11], we were able to explore the underlying factor structures of the mother–daughter SAWMS and select subscale items to retain maximum precision while maintaining adequate content coverage, resulting in three refined subscales (i.e., maternal control, maternal autonomy support, and maternal collaboration) in Study 1. In this study, we used another sample of college-aged cisgender young women to first substantiate the factor structure of the mother–daughter SAWMS established in Study 1, using CFA. We then used a sub-sample of these participants to assess each subscale’s test–retest reliability. Based on these results, we conducted another CFA of the two-factor model, after the exclusion of the maternal collaboration subscale.

## Method

### Participants and procedure

Participants were 439 US-raised cisgender young women aged between 18 and 29 (*M* = 20.28; *SD* = 1.40) and enrolled at the public research university where data collection took place. The original sample consisted of 468 participants; we excluded 16 who did not complete most of the survey measures and 13 who indicated that they did not have a mother or mother figure in their lives. The sample is ethnically diverse: 162 (36.90%) Latinx Americans, 135 (30.39%) Asian and Pacific Islander Americans, 76 (17.31%) European Americans, and 66 (15.03%) who identified as biracial or other ethnic minorities (e.g., African American, Middle Eastern American). In terms of mothers’ educational attainment, around half of the mothers had a high school or lower degree (*n* = 240; 54.67%), 56 (12.76%) had an Associate’s degree, 88 (20.04%) had a Bachelor’s degree, and 46 (10.48%) had a Master’s or higher degree. In terms of living arrangements, the majority of the participants (*n* = 296, 67.43%) were living with their mothers at the time of survey. Among those (*n* = 135) who indicated that they were not staying with their mothers, 56 (37.04%) said they had interactions with their mothers on a daily basis, 60 (44.44%) indicated interactions with their mothers on a weekly basis, and 19 (14.07%) interacted with their mothers on a monthly basis or less.

Similar to Study 1, data were collected at the Human Subject Pool at the public research university in Southern California during Fall 2020. Women who were over 18 and enrolled in the subject pool completed the study survey in exchange for half an extra class credit. The online survey included the mother–daughter SAWMS as well as measures of maternal pressure to be thin, perceptions of body size, body dissatisfaction, and mother–daughter relationships. In Spring 2021, six months after the first survey, participants who had left contact information (*N* = 420) were followed up with a similar survey via email. Independent *t*-tests of the baseline data indicated that those who participated in the follow-up survey (*N* = 145; 34.52% participation rate) did not significantly differ from those who did not participate in terms of age [*t*(418) = 0.16, *p* = 0.87], BMI [*t*(435) = − 1.49, *p* = 0.14], maternal control [*t*(437) = 0.54, *p* = 0.30], maternal collaboration [*t*(437) = 0.136, *p* = 0.09], maternal autonomy support [*t*(437) = 0.42, *p* = 0.34], and body dissatisfaction [*t*(415) = 0.42, *p* = 0.68]. For the current study, we focused on the mother–daughter SAWMS.

### Data analysis

We first conducted a CFA using maximum likelihood estimation to examine the three-factor structure of the mother–daughter SAWMS. To assess model fit, we selected the Comparative Fit Index (CFI), the Tucker-Lewis Index (TLI), the Root Mean Square Error of Approximation (RMSEA), and the Standardized Root Mean Square Residual (SRMR) to supplement the Chi-Square test. For CFI and TLI, values above 0.90 and 0.95 are generally indicative of adequate and good fit; values less than 0.08 and 0.05 for RMSEA are generally indicative of adequate and good fit; a value less than 0.08 for SRMR generally reflects good fit [6, 26].

Before examining test–retest reliability, we screened for missing data. Little’s MCAR (missing completely at random) test suggested that data across two waves were missing completely at random, *X*^*2*^ (205) = 192.25, *p* = 0.73. Thus, we used multiple imputation with 20 times repetition (Rubin, 1996) in SPSS 28 to deal with missing data, after which we estimated an intraclass correlation coefficient (ICC) for each subscale using an absolute-agreement, 2-way mixed-effects model [29]. ICC values less than 0.50 reflect poor reliability,values between 0.50 and 0.75 indicate moderate reliability; values larger than 0.75 reflect good reliability [29]. Finally, we conducted another CFA using maximum likelihood estimation to examine the two-factor structure of the mother–daughter SAWMS, with the exclusion of the maternal collaboration subscale.

## Results

### CFA results of the three-factor model

Fit indices indicated that the three-factor model had adequate fit to the data, *X*^*2*^(41) = 164.71, *p* < 0.001; CFI = 0.946; TLI = 0.928; RMSEA = 0.083, 90% CI = [0.070, 0.096]; SRMR = 0.066. All standardized factor loadings were significant at the *p* < 0.001 level and ranged from 0.72 to 0.85 for *maternal control*, 0.72 to 0.79 for *maternal autonomy support*, and 0.73 to 0.80 for *maternal collaboration*. Given the model only had adequate fit, we requested modification indices, which indicated that the item “My mom encourages me to be the weight I want to be” might cross-load on *maternal control*. Given that it is purely exploratory than theoretical, however, we decided to keep this item. We wanted to point out this possibility so that future researchers can make further explorations. Factor loadings are presented in Table 3.

**Table 3:** CFA loadings of subscale items for mother–daughter SAWMS

Items	Unstandardized loadings (*SE*)	Standardized loadings (*SE*)
*Maternal Control*		
My mom wants me to be a certain body weight	1.00 (0.00)	0.82 (0.02)
My mom tries to make me eat more or less because of my body weight	0.86 (0.05)	0.72 (0.03)
My mom attempts to control my eating portions because of my weight	0.81 (0.05)	0.77 (0.02)
My mom nags at me about how I am managing my weight	1.05 (0.05)	0.85 (0.02)
*Maternal Autonomy Support*		
My mom is very supportive of how I manage my weight	1.00 (0.00)	0.76 (0.03)
My mom encourages me to be the weight I want to be	0.97 (0.07)	0.72 (0.03)
My mom just wants me to be happy about my body weight	0.97 (0.06)	0.79 (0.02)
My mom feels like my body weight is my own business	0.99 (0.07)	0.72 (0.03)
*Maternal Collaboration*		
My mom works with me on my body weight issues	1.00 (0.00)	0.75 (0.03)
My mom works together with me in managing my body weight	1.06 (0.07)	0.80 (0.03)
My mom helps me to be the weight I want to be	0.98 (0.07)	0.73 (0.03)

All standardized factor loadings were significant at the *p* < 0.001 level

### Test–retest reliability results

ICC estimates and their 95% CI were calculated using SPSS version 28 based on a single-measurement, absolute-agreement, 2-way mixed-effects model [29]. For the maternal control subscale, the estimated ICC was 0.88, 95% CI = [0.86, 0.90], indicating high stability. For the maternal autonomy support subscale, the estimated ICC was 0.79, 95% CI = [0.75, 0.82] , indicating moderate to high stability. For the maternal collaboration subscale, the estimated ICC was 0.47, 95% CI = [0.39, 0.54] , indicating poor stability.

### CFA results of the two-factor model

Given the low ICC for maternal collaboration (i.e., 0.47), we conducted a two-factor CFA with only the maternal control and maternal autonomy support subscales. Fit indices indicated that the two-factor model had adequate fit to the data, *X*^*2*^(19) = 129.78, *p* < . 001; CFI = 0.955; TLI = 0.933; RMSEA = 0.093, 90% CI = [0.078, 0.108]; SRMR = 0.053. All standardized factor loadings were significant at the *p* < 0.001 level and ranged from 0.69 to 0.84 for *maternal control*, 0.69 to 0.81 for *maternal autonomy support*.

Based on the fit indices of the two-factor model and other empirical evidence, we decided to exclude the maternal collaboration subscale in the final version of the SAWMS. First, the ICCs for maternal collaboration (i.e., 0.47) were far lower than ideal. Second, the EFA in Study 1 indicated that the maternal collaboration subscale only accounted for an additional 6.85% of the variance. Third, results from IRT calibration in Study 1 showed that the three items indicating maternal collaboration were not significantly different in their ability to discriminate among respondents. Finally, the correlation between maternal collaboration and body dissatisfaction was near 0 (*r* = 0.03, *p* = 0.53). Thus, the following study examining psychometric properties only focused on the maternal control and maternal autonomy support subscales.

## Study 3: Different types of validities and associations with body dissatisfaction

In this study, we used the same sample as study 2 (*N* = 439) to examine the psychometric properties of the two subscales of maternal control and maternal autonomy support. Specifically, we examined convergent validity, divergent validity, discriminant validity, and incremental validity (e.g., predicting body dissatisfaction). For convergent validity, we hypothesized that maternal control would be significantly and positively correlated with BMI, maternal pressure to be thin, and body dissatisfaction; maternal autonomy support would be significantly and positively correlated with perceived maternal expectation of larger body size. For divergent validity, we hypothesized that maternal control would be significantly and negatively correlated with perceived maternal expectation of larger body size and maternal autonomy support; maternal autonomy support would be significantly and negatively correlated with BMI, maternal pressure to be thin, and body dissatisfaction. For discriminant validity, we examined the correlations between our subscales and ethnic commitment, given that the extent to which one is connected to or involved in their ethnic group presumably would not be associated with mother–daughter relationship dynamics in weight management. Thus, we hypothesized that the correlations between ethnic commitment and the two subscales would be near zero.

For hierarchical regression analyses examining incremental validity of the subscales (i.e., predicting body dissatisfaction), we hypothesized that (1) our newly developed subscales of maternal control and maternal autonomy support in weight management would be significantly associated with body dissatisfaction over and above the effect of the existing measure of maternal pressure to be thin, given that the shared agency subscales measure the specific mother–daughter dynamics in weight management whereas the subscale of the Sociocultural Attitudes Towards Appearance Questionnaire-4-Revised (SATAQ-4R; [41]) assessing maternal pressure to be thin is more about general maternal attitudes, (2) maternal control in weight management would be positively associated with daughters’ body dissatisfaction, and (3) maternal autonomy support in weight management would be negatively associated with daughters’ body dissatisfaction.

### Measures

#### Demographics

Participants were asked to indicate their age, sex assigned at birth, gender, ethnicity, and their mothers’ educational attainment. They were also asked to report their weight and height, from which BMI was calculated (kg/m^2^).

#### Self-perception of body size

Widely used in body image studies, the figure rating scale was used to assess participants’ self-perception of body size [45]. Nine schematic figures of female bodies (1 = *Very thin*, 9 = *Very large*) were presented to participants. They were asked to select the figure that best reflects their current body shape. In previous studies, participants’ ratings were used to compare with their BMI or ideal body shape to calculate a discrepancy, which was then used to indicate body image disturbance [40] and body dissatisfaction [31]. The scale was strongly correlated with BMI in a sample of college students (*r* = 0.67, *p* < 0.001; [20]). For the current sample, the scale was highly correlated with BMI (*r* = 0.79, *p* < 0.001) and weight (*r* = 0.74, *p* < 0.001).

#### Perceived maternal expectation of body size

The same figure rating scale assessing participants’ self-perception of body size [45] was adapted to measure their perceived maternal expectation of body size. They were asked to select the Fig. (1 = *Very thin*, 9 = *Very large*) that best represented their perception of how their mothers expected them to look with regard to body size. A large number reflects perceived maternal expectation of a *larger* body size. For the current sample, the scale was significantly and negatively correlated with maternal pressure to be thin (*r* = − 0.23, *p* < 0.001) and was significantly and positively correlated with BMI (*r* = 0.31, *p* < 0.001).

#### Ethnic commitment

The three-item ethnic commitment subscale of the Multigroup Ethnic Identity Measure-Revised (MEIM-R; [39]) was used to assess participants’ ethnic commitment, which was used to examine the discriminant validity of our newly developed subscales. One sample item was “I have a strong sense of belonging to my ethnic group.” Participants were asked to indicate the extent to which they agreed or disagreed with the items on a 6-point Likert scale (1 = *Strongly disagree*, 6 = *Strongly agree*). Average scores were calculated, with higher scores reflecting greater levels of ethnic commitment. Alpha for the current sample was 0.90.

#### Maternal pressure to be thin

Maternal pressure to be thin was measured by a revised version of the family subscale of the Sociocultural Attitudes Towards Appearance Questionnaire-4-Revised (SATAQ-4R; [41]). The original four items aim to assess family members’ attitudes towards young women’s appearance and body image. We changed the wording “family members” to “my mother” in order to measure maternal pressure to be thin toward daughters. One sample item was “my mother encourages me to get thinner.” Participants were asked to indicate the extent to which they agreed or disagreed with the items on a 6-point Likert scale (1 = *Strongly disagree*, 6 = *Strongly agree*). Average scores were used to indicate maternal pressure to be thin, with higher scores reflecting greater levels of pressure. Alpha for the current sample was 0.95.

#### Mother–daughter SAWMS

The eight-item Mother–Daughter SAWMS was used to assess maternal control and maternal autonomy support in daughters’ weight management. Participants were asked to indicate the extent to which they agreed or disagreed with the statements about how their mothers worked with them in weight management on a 6-point Likert scale (1 = *Strongly disagree*, 6 = *Strongly agree*). In the current sample, alphas were 0.87 and 0.84 for the maternal control subscale and the maternal autonomy support subscale, respectively. See Tables 2 and 3 for subscale items.

#### Body dissatisfaction

The nine-item body dissatisfaction subscale of the Eating Disorder Inventory-2 (EDI-2; [21]) was used to assess body dissatisfaction. Participants were asked to indicate the extent to which the statements reflecting body dissatisfaction applied to them in the past year on a 6-point Likert scale (1 = *Never*, 6 = *Always*). Scores were averaged to indicate body dissatisfaction, with higher score reflecting greater levels of body dissatisfaction. One sample item was “I think that my thighs are too large.” The subscale was used widely in previous studies [3, 48]. Alpha for the current sample was 0.82.

### Data analysis

To examine convergent and divergent validities, we performed bivariate correlations between each subscale and daughters’ BMI, self-perception of body size, perceived maternal expectation of body size, maternal pressure to be thin, and body dissatisfaction. To examine discriminant validity, we performed bivariate correlations between each subscale and ethnic commitment. Finally, to examine incremental validity, we used hierarchical linear regression to explore the associations between the two subscale of interests (i.e., maternal control and maternal autonomy support) and daughters’ body dissatisfaction over and above the effect of maternal pressure to be thin. Given our theoretical interests, we decided to control for BMI and daughters’ self-perception of body size.

Before conducting the hierarchical regression analysis, we examined relations between demographic variables (e.g., age, maternal educational attainment, ethnicity) and body dissatisfaction to see if we need to include other control variables. An One-way Analysis of Variance indicated that there were no significant differences in body dissatisfaction across main ethnic groups, *F*(2, 355) = 0.49, *p* = 0.61. Neither age (*r* = 0.06, *p* = 0.21) nor maternal educational attainment (*r* = − 0.08, *p* = 0.12) was significantly associated with daughters’ body dissatisfaction; we thus did not include them as covariates. We also screened for missing data. Most measures had either no or less than 2 percent (*n* < 7) of missing values; body dissatisfaction had the most missing values (*n* = 22; 5.01%). Little’s MCAR (missing completely at random) test suggested that data were missing completely at random, *X*^*2*^ (24) = 21.59, *p* = 0.60. We thus used multiple imputation with 20 times repetition (Rubin, 1996) in Stata 15 to deal with missing data, after which the hierarchical regression analysis was performed using multiple imputation estimation. In Step 1, we entered BMI, daughters’ self-perception of body size, and maternal pressure to be thin. In Step 2, we entered maternal control and maternal autonomy support. The procedures were automatically done in Stata, which involve the combination of the estimates from each of the 20 imputed datasets to obtain one final set of inferential statistics.

## Results

### Bivariate correlations assessing convergent, divergent, and discriminant validities

Maternal control was significantly, positively, and weakly correlated with daughters’ BMI (*r* = 0.21, *p* < 0.001), strongly correlated with maternal pressure to be thin (*r* = 0.68, *p* < 0.001), but significantly, negatively, and weakly correlated with perceived maternal expectation of larger body size (*r* = − 0.15, *p* = 0.002). Maternal autonomy support was significantly, negatively, and weakly correlated with daughters’ BMI (*r* = − 0.18, *p* < 0.001), strongly correlated with maternal pressure to be thin (*r* = − 0.54, *p* < 0.001), but significantly, positively, and weakly correlated with perceived maternal expectation of larger body size (*r* = 0.21, *p* < 0.001). In terms of daughters’ body dissatisfaction, maternal control was significantly, positively, and moderately correlated with body dissatisfaction (*r* = 0.37, *p* < 0.001); maternal autonomy support was significantly, negatively, and moderately correlated with body dissatisfaction (*r* = − 0.34, *p* < 0.001). In terms of discriminant validity, the correlation between ethnic commitment and maternal control was near 0 (*r* = 0.09, *p* = 0.07); so was the correlation between ethnic commitment and maternal autonomy support (*r* = 0.03, *p* = 0.52) Correlation coefficients among main variables are presented in Table 4.

**Table 4:** Descriptive Statistics and Correlations Among Main Variables

	*M* (*SD*)	2	3	4	5	6	7	8
1. BMI	23.95 (5.34)	0.79***	0.31***	0.40***	0.21***	− 0.18***	0.07	0.34***
2. Self-perception of body size	5.19 (2.01)	–	0.48***	0.44***	0.23***	− 0.20***	0.01	0.45***
3. Maternal expectation of body size	4.09 (1.51)		–	− 0.23***	− 0.15**	0.21***	− 0.01	0.07
4. Maternal pressure to be thin	3.02 (1.65)			–	0.68***	− 0.54***	0.10*	0.39***
5. Maternal control	2.64 (1.26)				–	− 0.46***	0.09	0.37***
6. Maternal autonomy support	4.04 (1.14)					–	0.03	− 0.34***
7. Ethnic commitment	3.49 (1.00)						–	− 0.05
8. Body dissatisfaction	3.53 (0.92)							–

*p < 0.05, **p < 0.01, *** *p* < 0.001

### Hierarchical linear regressions predicting daughters’ body dissatisfaction

The full two-step model was significant and explained 28.8% of the variance in daughters’ body dissatisfaction [*F*(5, 426.7) = 34.77, *p* < 0.001; *R*^2^ = 0.296, Adj. *R*^2^ = 0.288]. Step 1 variables accounted for significant variance in daughters’ body satisfaction [*F*(3, 424.0) = 45.51, *p* < 0.001, *R*^2^ = 0.248, Adj. *R*^2^ = 0.243]. Daughters’ self-perception of body size [*B*(*SE*) = 0.18 (0.03), *ß* = 0.39, *p* < 0.001] and maternal pressure to be thin [*B*(*SE*) = 0.13 (0.03), *ß* = 0.24, *p* < 0.001] were significant predictors whereas BMI was not. The addition of the two mother–daughter SAWMS subscales in Step 2 significantly explained 4.8% of the additional variance in daughters’ body satisfaction [Δ*R*^2^ = 0.048; Δ*F*(2, 424.7) = 14.20, *p* < 0.001, *f*^2^ = 0.05]. Daughters’ self-perception of body size [*B*(*SE*) = 0.19 (0.03), *ß* = 0.41, *p* < 0.001], maternal control [*B*(*SE*) = 0.15 (0.04), *ß* = 0.20, *p* = 0.001], and maternal autonomy support [*B*(*SE*) = − 0.14 (0.04), *ß* = − 0.17, *p* = 0.001] were significant predictors whereas BMI and maternal pressure to be thin [*B*(*SE*) = − 0.003 (0.04), *ß* = − 0.01, *p* = 0.94] were not. Thus, maternal control and maternal autonomy support in weight management explained significant variance in daughters’ body dissatisfaction over and above the effect of maternal pressure to be thin. Controlling for all other variables, maternal control in weight management was a significant and positive predictor of body dissatisfaction; maternal autonomy support in weight management was a significant and negative predictor of body dissatisfaction.

## Discussion

The current paper described the development and validation of the mother–daughter shared agency in weight management scale (SAWMS) and examined its associations with daughter’s body dissatisfaction. To our knowledge, this is the first study to apply the novel shared agency framework [11] to the investigation of body image among young women, examining the specific ways in which mothers work with their daughters in weight management. Combining results from EFA and IRT, we identified three mother–daughter dynamics in weight management—maternal *control*, maternal *autonomy support*, and maternal *collaboration*. Given that the test–retest reliability for the maternal collaboration subscale was poor, however, we did not proceed to evaluate its psychometric properties. For the remaining two subscales, our analyses showed that maternal *control* was a significant and positive predictor of daughters’ body dissatisfaction, whereas maternal *autonomy support* was a significant and negative predictor. As such, mother–daughter shared agency in weight management (i.e., maternal autonomy support) may be beneficial for the development of positive body image among young women, whereas mother–daughter non-shared agency in weight management (i.e., maternal control) may have detrimental effects for daughters’ body dissatisfaction.

In Study 1, we explored the factor structure of the mother–daughter SAWMS. Interestingly, the EFA revealed that items indicating maternal support (e.g., *my mom is very supportive of how I manage my weight*) and maternal accommodation (e.g., *my mom feels like my body weight is my own business*) loaded on the same factor, which we renamed as maternal *autonomy support*. Moreover, given the unbalanced numbers of items among the three subscales resulting from EFA, we used IRT to refine each subscale, selecting items that retained maximum precision and information while also maintaining adequate content coverage. For the maternal *control* subscale, item 5 (i.e., my mom talks with me about my weight) and item 7 (i.e., my mom negotiates with me on how I manage my body weight) were initially theorized to load on maternal *collaboration*, as mother–daughter talk and negotiations might reflect more of joint endeavors than maternal direction and control. Given that both items also had relatively lower EFA loadings, discrimination parameters, and correlations with subscale compared to the other four items, we decided to remove them. In the maternal *autonomy support* subscale, both item 13 (i.e., my mom can let go of her own issues with my body weight) and item 14 (i.e., my mom does not feel responsible for managing my weight) had moderate item discrimination and very low item information, whereas the other four items all had very high discrimination, information, and item-subscale correlation. We thus excluded them to achieve higher precision.

Although parental *support* and parental *accommodation* were thought to be distinct constructs in previous studies examining shared agency in educational pursuit [11], the fact that items of maternal autonomy support and maternal accommodation loaded on the same factor suggests that shared agency between youth and parents might manifest differently in weight management. Indeed, educational pursuit is more of an external task, whereas body image is more of an internal social identity vulnerable to stigma and judgment [19]. This might be why maternal accommodation in weight management was perceived as a way of providing support by daughters in the current study. Previous studies suggested that maternal comments on daughters’ body image, even when delivered with benign intentions or in forms of encouragement to lose weight, can be maladaptive [4, 27]. Thus, in a society rife with judgment toward female bodies, maternal accommodation (i.e., taking a step back) might be interpreted as a form of support. Future research should examine how parent-youth shared agency functions in other domains and how parental support and accommodation are different from or similar to each other.

In Study 2, we used a different sample to finalize the factor structure of the scale. We also used six-month follow-up data from a subsample of participants in the study to examine the test–retest reliability of each subscale. Results from CFA indicated that the three-factor structure of the mother–daughter SAWM had adequate model fit. However, unlike the subscales of maternal autonomy support and maternal control, both of which showed high stability in Study 2 sample, the stability of the maternal collaboration subscale across six months was low (i.e., 0.47), indicating poor test–retest reliability. Without the maternal collaboration subscale, results from the CFA indicated that the two-factor structure of the mother–daughter SAWM also had adequate model fit. Considering that the three items indicating maternal collaboration did not have significantly different ability to discriminate among respondents and that the subscale had almost no correlation with body dissatisfaction (0.03), we excluded this subscale. However, given the theoretical underpinning of parent-youth collaboration in life pursuit [11], the high internal consistency of the maternal collaboration subscale in Study 1 (e.g., alpha > 0.80), and the large amount of missing data in the follow-up sample assessing test–retest reliability, future scholars should further explore this construct of maternal collaboration in weight management and its validity and reliability (e.g., test–retest reliability across two or three weeks).

In Study 3, we examined different types of validities of the maternal autonomy support and maternal control subscales as well as their associations with daughters’ body dissatisfaction. Correlations between the two subscales and other measures (e.g., BMI, maternal pressure to be thin, maternal expectation of body size, and ethnic commitment) were all in expected directions (see Table 4), indicating that the two subscales in the sample had excellent convergent, divergent, and discriminant validities. Results from regression analyses suggested that maternal *control* was significantly and positively associated with body dissatisfaction, whereas maternal *autonomy support* was significantly and negatively associated with body dissatisfaction. These results were not only found in the correlational analyses at the bivariate level but also remained consistent in the hierarchical linear regression analysis when BMI, daughters’ self-perception of body size, maternal pressure to be thin were controlled, which indicated that maternal control and maternal autonomy support explained significant variance in daughters’ body satisfaction over and above the effects of these variables.

Importantly, our subscales explained significant variance in body dissatisfaction over and above the effect of the validated measure assessing maternal pressure to be thin, suggesting that the specific ways in which mother work with daughters may provide more nuances in understanding young women’s body dissatisfaction than general maternal attitudes towards body image and weight. Although the subscales only explained 4.8% additional variance of daughters’ body dissatisfaction, with a small-to-medium effect size (i.e., *f*^2^ = 0.05), this magnitude aligns with statistics reported in existing literature examining the role of maternal influence on body dissatisfaction among youth and emerging adults (e.g., [36, 42, 49]). Future work should compare the influence of shared agency with other relational contributors to body dissatisfaction.

Thus, our findings pinpoint the importance of the *specific* mother–daughter relationship dynamics in shaping daughters’ weight management. Although the role of mother–daughter relationships in predicting body dissatisfaction has been extensively explored, our study suggests that the specific ways in which mothers and daughters work together to manage daughters’ weight is a new frontier that has explanatory power in the prediction of body dissatisfaction. Mothers could offer more autonomy support and less control when it comes to daughters’ weight management, fostering mother–daughter shared agency for the development of positive body image and perceptions among daughters. Future work can further explore the ways in which shared agency may develop over time within the broader context of mother–daughter relationships, as well as how individual differences and daughters’ age shape such processes.

In addition, in light of previous developmental research regarding the relation between parental control and autonomy support [43], results from both correlation and regression analyses suggest that, at least in the domain of weight management, maternal *control* and maternal *autonomy support* (*r* = − 0.46, *p* < 0.001) are distinct constructs rather than opposite ends on a single continuum. Indeed, maternal control on daughters’ weight may reflect more of mothers’ concern than the suppression of volitional functioning; less maternal control thus might not necessarily equate more autonomy support when it comes to weight management. Scholars should further examine how these two parenting practices affect body dissatisfaction, both uniquely and interactively.

Although the current study is the first to draw on the framework of shared agency in understanding young women’s body image, the findings should be interpreted in light of some limitations. First, due to our cross-sectional design, it could be that daughters’ body dissatisfaction affects how they perceive their mothers’ behaviors. For instance, daughters who are more dissatisfied about their body may be more likely to perceive mothers’ behaviors as controlling. Thus, future studies should utilize longitudinal designs to infer causal relations between maternal behaviors and daughters’ body dissatisfaction. Second, we only focused on the effects of how mothers work with daughters in weight management, thus leaving other important factors unexamined, such as the roles of fathers, social media, and peer relationships. We understand that weight stigma toward and body image concerns of women are complex social issues; thus our intention is never to blame mothers but to examine what mother–daughter relationship dynamics can potentially promote healthy body image among young women. Therefore, future studies should examine the roles of fathers as well as social media and peer relationships in shaping young women’s body dissatisfaction. Moreover, we focused on body dissatisfaction among college-aged emerging adults; future studies should examine the effect of mother–daughter/parent–child shared agency on body image issues among other age groups, such as adolescents and children, for whom parental influences may play more significant roles. Further, although daughters’ self-reports are likely to capture memories that are the most salient (and thus influential) to them in terms of mothers’ influence on weight management, it would be instructive to compare behavioral observations of mealtime encounters and mothers’ own reports of SAWMS scores in future studies to provide additional evidence of validity.

## Conclusion

Across three studies, we developed and validated the mother–daughter Shared Agency in Weight Management Scale (SAWMS). We identified two ways (i.e., control and autonomy support) whereby mothers work with daughters in weight management and examined their associations with daughters’ body dissatisfaction. Maternal control in weight management is associated with higher levels of body dissatisfaction among daughters, whereas maternal autonomy support in weight management is associated with lower levels of body dissatisfaction. These findings add nuances to the existing literature on the important role of mothers in shaping daughters’ body image. We hope that future researchers will apply the SAWMS in their research and test the processes we have identified in other samples and relationships (e.g., mother-son dyads, father-daughter dyads) when studying body image.

## Data Availability

The data are available from the corresponding author on reasonable request.
